# Shearing-force injury of a kidney transplant graft during cesarean section: a case report and review of the literature

**DOI:** 10.1186/s12882-019-1281-6

**Published:** 2019-03-18

**Authors:** Catherine E. Gordon, Vasiliki Tatsis

**Affiliations:** Department of Obstetrics & Gynecology, University of California, Irvine, 333 City Blvd, West - Suite 1400, Orange, CA 92868 USA

**Keywords:** Kidney transplant, Cesarean section, Incision, Injury

## Abstract

**Background:**

With an increasing number of reproductive-aged women undergoing renal transplantation coupled with improved fertility post-transplantation, more women are becoming pregnant with a kidney transplant in place. This leads to increased risk of perinatal complications such as pre-eclampsia, gestational diabetes, preterm delivery and Cesarean section. Given that kidney transplants are often placed extra-peritoneally in the iliac fossa, there is also a risk of damage to the transplanted kidney at the time of Cesarean section.

**Case presentation:**

We present a case of shearing-force injury to a transplanted kidney at the time of repeat Cesarean section due to adherence of the organ to the overlying fascia. This is the first known case of an injury by this mechanism.

**Conclusion:**

Pre-operative planning with organ mapping and incision planning is imperative, with consideration for a vertical midline incision to avoid direct or shearing forces on the transplant kidney. Preoperative collaboration with the Transplant Surgery team is also important so they are available in case of emergency or need for intraoperative consultation.

## Background

With an ever-increasing number of kidney transplantations each year and prolonged life expectancy after transplant in patients with end stage renal disease (ESRD), more women of reproductive age are becoming pregnant. It is well documented that pregnancy carries increased antepartum risks in transplant patients and leads to increased risk of Cesarean section. Kidney transplants are typically placed pre-peritoneally in the lower abdomen, which is in the operative field of a Pfannenstiel incision at the time of Cesarean section. We present the case of a patient with an adherent kidney transplant in the lower right abdomen who underwent repeat Cesarean section with indirect shearing-force injury caused to the kidney upon routine elevation of the overlying fascia. This is the first known case that describes injury by this mechanism, which suggests the rarity of this complication and may also be a result of under-reporting bias. We propose that a midline vertical skin incision can be considered in the kidney transplant patient undergoing a Cesarean section, in addition to pre-delivery collaboration and organ mapping with the Transplant Surgery team.

Approximately 33,000 people underwent organ transplantation last year, of which 19,000 received a kidney transplant [1]. Women aged 18–49 comprised 39% of those kidney transplant recipients, totaling approximately 7500 women. Patients with ESRD have decreased fertility, especially in the setting of dialysis, given hypothalamic-gonadal dysfunction [2]. Renal transplantation, however, can reverse this trend with a return of fertility in as little as one month [3]. With improved fertility in reproductive aged patients, obstetricians are seeing more solid organ transplant patients. Pregnancy carries increased risk in transplant patients, with increased incidence of pre-eclampsia, gestational diabetes, preterm delivery, fetal growth restriction, and Cesarean section [3–6]. An international meta-analysis demonstrated a 57% risk of Cesarean section in the setting of kidney transplantation, compared to the 32% national average [4]. A national cohort study conducted in Norway demonstrated an adjusted four-fold increased rate of Cesarean section in kidney transplant patients compared to controls [5].

## Case presentation

A 35-year-old G4P2012 presented to labor and delivery at full term in early labor with spontaneous rupture of membranes. She had undergone living donor kidney transplant 8 years prior for ESRD suspected secondary to IgA nephropathy versus post-streptococcal glomerulonephritis. She was maintained on Prograf and prednisone throughout her pregnancy with a stable baseline serum creatinine 1.2 mg/dL. Obstetrical history was significant for two prior uncomplicated Cesarean deliveries, both prior to her kidney transplantation. It was unclear from her records whether she had undergone counseling regarding pregnancy post-transplant. She declined a of trial of labor after Cesarean section despite counseling on the risk of injury to the transplanted kidney given its location in the operative field, which was confirmed on transabdominal ultrasound prior to the case.

**Fig. 1:**
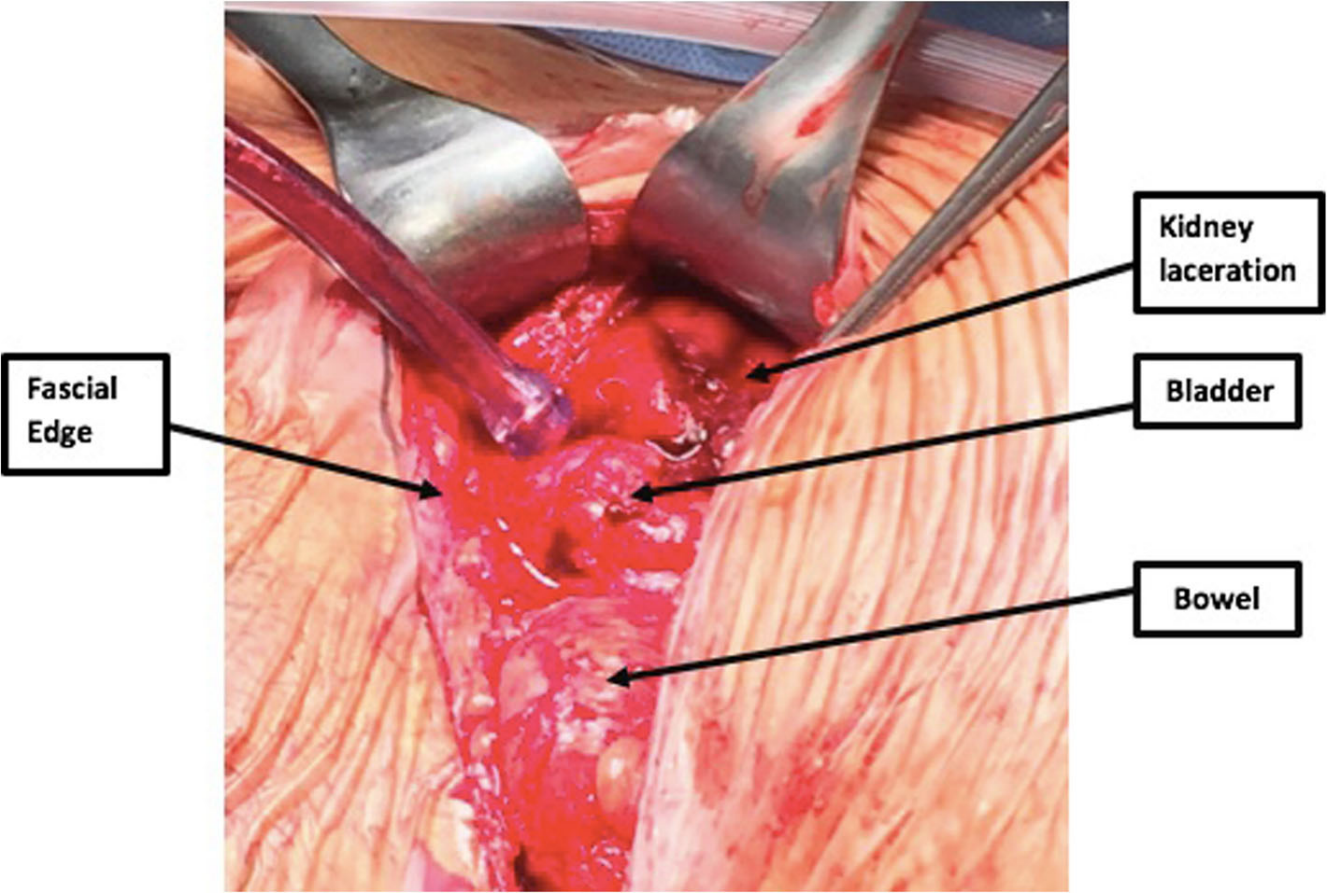
Intra-operative view of kidney transplant laceration

The patient was taken to the operating room for and indicated repeat Cesarean section. Routine dissection was performed down to the level of the fascia through a Pfannenstiel incision and the fascia was carefully transected horizontally in the usual fashion. When the superior fascial edge was lifted for traction to allow for dissection from the underlying rectus muscle, brisk pulsatile bleeding was noted. The transplanted kidney was noted to be densely adherent to the overlying anterior abdominal wall, and avulsion of a portion of the transplanted kidney was confirmed (Fig. 1). Direct pressure was applied to the organ while the Trauma Surgery team was consulted. The avulsion was repaired using 3–0 pledgetted Prolene sutures in interrupted horizontal mattress fashion and using Evarrest compound. At this time, the decision was made to extend the skin incision in the vertical midline for improved access to the uterus given the concern for further shearing forces to the exposed transplanted kidney in the process of delivering the baby. The rest of the case was uneventful. No blood product transfusion was necessary. After the transplanted kidney was repaired, a viable male infant with Apgars of 8 and 9 weighing 2460 g was delivered.

Postoperative serum creatinine levels remained stable between 1.1–1 .3mg/dL and the patient maintained adequate urine output. Renal ultrasound immediately postpartum and 1 month postpartum demonstrated normal arterial and venous flow with no evidence of ischemia or perinephric hematoma.

## Discussion and conclusions

While many studies have cited the increased risks of pregnancy in transplant patients, there is a paucity of literature describing the risk of damage to the transplanted kidney and management of this patient population during Cesarean section. There are increased rates of Cesarean section in kidney transplant patients, commonly due to fetal malpresentation or non-reassuring fetal heart rate tracings in indicated preterm deliveries typically done for hypertensive disorders or fetal growth restriction. Careful delivery planning with particular attention to skin and fascial incisions is imperative.

A multidisciplinary team should be involved in the care and delivery planning of a patient with previous kidney transplant. The Transplant Surgery team should be present or readily available at the time of delivery, particularly if Cesarean section is indicated. Ideally, the Transplant Surgery team should be consulted in the antepartum period to assist with surgical planning, with consideration for pre-operative imaging to confirm transplant location.

While several studies comment on the importance of avoiding injury to graft, no specific recommendations exist to on how to prevent this potentially devastating complication [6]. One case report describes injury to a pelvic transplanted kidney due to accidental transection. While careful dissection may have mitigated injury in that case, it most likely would not have prevented organ injury in our case. Adhesions between the kidney and overlying fascia and underlying peritoneum led to avulsion simply by lifting the fascia for traction. A midline vertical skin incision could have avoided this complication by minimizing the opposing forces that led to avulsion. A vertical skin incision would also mitigate fascial dissection and closure in close proximity to this vascular and delicate organ.

There is limited data on the appropriate skin incision at the time of Cesarean section for kidney transplant patients, with only one retrospective cohort describing incision type. Fang et al. describe 22 patients with single kidney transplant or simultaneous pancreas and kidney transplants. Sixteen deliveries were by Cesarean section, 15 of which were via a Pfannenstiel incision [7]. Two cases were complicated by cystotomy in the simultaneous pancreas and kidney group, while all single kidney transplant Cesarean sections via Pfannenstiel incision were uncomplicated. The authors argue that use of a Pfannenstiel incision is appropriate in kidney transplant patients. There are a number of other reports in the literature of uncomplicated Cesarean sections in renal transplant patients without description of incision type. Farr et al. describe 12 ultra-high-risk pregnancies, of which 8 underwent Cesarean section, all of which were uncomplicated except one which required hysterectomy due to placenta percreta [8]. Mohammadi et al. describe uncomplicated Cesarean sections in 28 out of 35 pregnancies in patients with kidney transplant [9]. Neither of these studies describe the incision type, but assuming routine Pfannenstiel incisions were made in some, if not most, of these patients, then the complications rate appears low.

Careful preparation for Cesarean section in kidney transplant patients is imperative. Providers should read the operative report from the transplant surgery to make themselves familiar with the anatomy and any potential complications, such as dense adhesions noted on previous abdominal entry. Providers should perform transabdominal ultrasound at a minimum to localize the kidney prior to surgery, either formally or at bedside. Providers can also consider MRI if ultrasound is limited or if better visualization is needed, such as assessing for abnormal placentation.

**Fig. 2:**
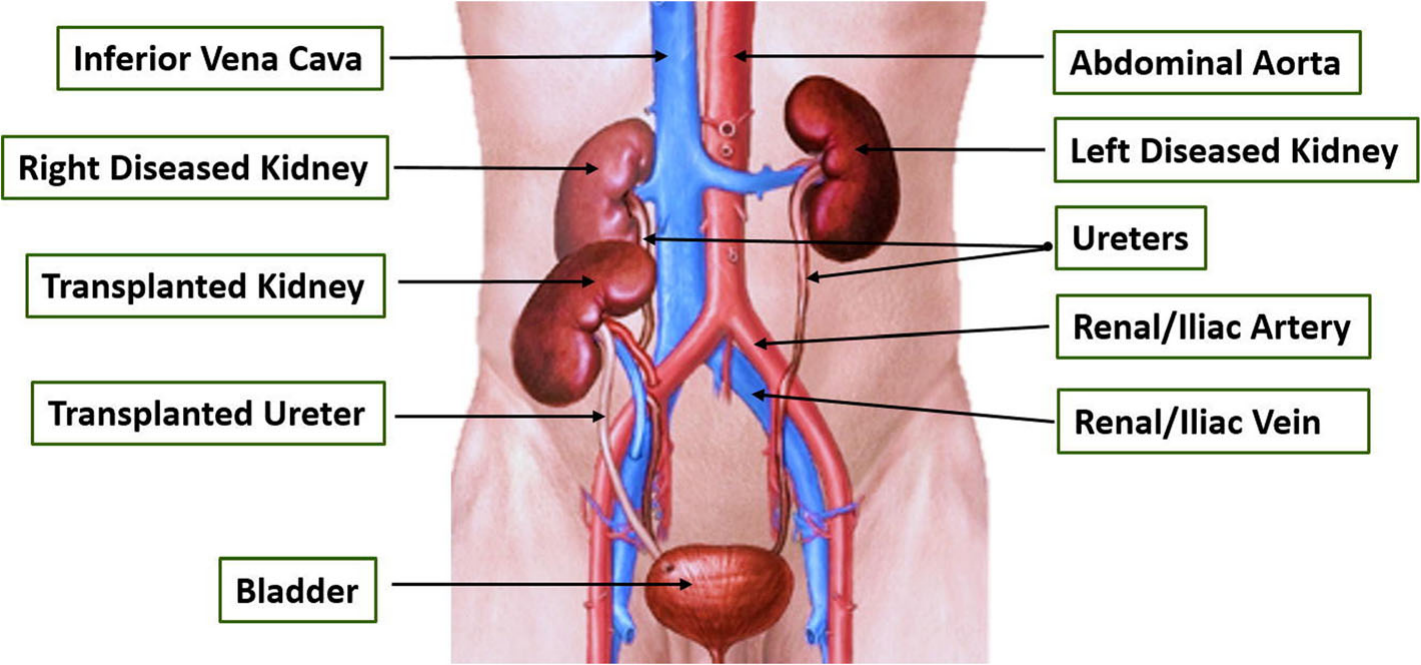
Anatomy of the renal system after kidney transplantation. *Figure reproduced from: Elizabeth Hollis E, Shehata M, Khalifa F, Abou El-Ghar M, El-Diasty T, El-Baza A. Towards non-invasive diagnostic techniques for early detection of acute renal transplant rejection: A review. Egyptian Journal Rad and Nucl Med. 2017 Mar; 48(1): 257–269*

Cesarean section in the setting of renal transplant should only be performed for obstetrical indications [10]. Availability of blood products should be confirmed and precise organ location with ultrasound should be performed prior to delivery via Cesarean section. Surgeons should collaborate with the Transplant Surgery team. It is also important to understand the anatomy of a transplanted kidney. Typically, the renal vessels are anastomosed to the iliac vessels, either the common iliac or external iliac artery and vein, and the ureter is connected to the bladder from the iliac fossa [11] (Fig. 2). A clear understanding of these anatomic changes can help decrease the risk of injury. If a transplanted kidney is injured during Cesarean section, profuse bleeding can occur and lead to maternal hemodynamic instability. Direct pressure should be applied to prevent massive blood loss, blood products should be administered as needed, and the Transplant or Trauma Surgery team should immediately be called for repair of the injury.

In conclusion, obstetric patients with a history of renal transplant require special attention at the time of delivery. With the transplanted kidney is often present in the pelvic extraperitoneal spaces, providers should identify the location of the transplant graft via ultrasound or MRI and carefully plan the incision. Providers can consider a vertical midline incision to avoid injury to the transplant organ, especially in the setting of previous surgeries with dense adhesions. Early pre-operative planning and close coordination between teams is imperative to prevent complications to the fetus, mother, and transplant kidney.

## Abbreviation

ESRD: end stage renal disease
